# Nursing homes during the COVID-19 pandemic: a scoping review of challenges and responses

**DOI:** 10.1007/s41999-021-00531-2

**Published:** 2021-06-16

**Authors:** Shamik Giri, Lee Minn Chenn, Roman Romero-Ortuno

**Affiliations:** 1 School of Medicine, Trinity College Dublin, Dublin, Ireland; 2 Discipline of Medical Gerontology, Mercer’s Institute for Successful Ageing (MISA), St James’s Hospital, 6th Floor, Dublin, 8 Ireland; 3 Global Brain Health Institute, Trinity College Dublin, Dublin, Ireland

**Keywords:** COVID-19, Nursing homes, Older adults, Pandemic, Mortality

## Abstract

**Introduction:**

COVID-19 has caused unprecedented challenges in nursing homes. In this scoping review, we aimed to describe factors that contributed to the spread and mortality of COVID-19 in nursing homes and provide an overview of responses that were implemented to try to overcome such challenges.

**Methods:**

The MeSH terms “Nursing homes” and “COVID-19” were searched in MEDLINE Ovid, and English language articles were retrieved that were published between 1 March 2020 and 31 January 2021. Article titles and abstracts were screened by two reviewers, and the results of included articles were grouped by themes.

**Results:**

The search retrieved 348 articles, of which 76 were included in the thematic review. 8 articles related to COVID-19 disease characteristics (e.g. asymptomatic transmission), 24 to resident-related factors (e.g. comorbidities, nutrition, cognition), 13 to facility characteristics (e.g. physical space, occupancy, for-profit status), 21 to staffing (e.g. staffing levels, staff-to-resident ratio, staff multi-employment), and 10 to external factors (e.g. availability of personal protective equipment, prevailing health and social care policies). In terms of responses, identified themes included widespread testing, isolation and cohorting of residents, staff protection and support, promotion of residents’ well-being, and technological innovations.

**Conclusion:**

COVID-19 exerted severe challenges on the nursing home population and its staff. Both internal and external factors predisposed nursing homes to an increased propensity of spread. Numerous strategies were employed to attempt to mitigate the negative impacts. Substantial learning occurred that may not only aid future pandemic preparedness but improve quality of care for nursing home residents at all times.

## Key summary points

**Aim:**

To describe factors that contributed to the spread and mortality of COVID-19 in nursing homes and provide an overview of responses that were implemented.

**Findings:**

COVID-19 exerted severe challenges on the nursing home population and its staff. Both internal and external factors predisposed nursing homes to an increased propensity of spread.

**Message:**

Substantial learning occurred that will lead to better pandemic preparedness and improve quality of care for nursing home residents at all times.

## Introduction

Nursing homes have been one of the largest hotspots for the dissemination and mortality of the COVID-19 pandemic throughout the year of 2020 [1]. Nursing homes are facilities purposefully built for the residential and/or nursing care of older people living with advanced physical and/or cognitive disabilities. Nursing home residents very often require care and support that is of intimate nature, which makes them vulnerable to contracting easily spreadable infections such as COVID-19 [2, 3]. Understanding the unique characteristics of the nursing home population is important because in some European countries, the population of nursing home residents is projected to increase by up to 127% by 2050 [4].

The proportion of COVID-19 deaths that occurred in nursing homes is high. In May 2020, it was estimated that about half of the COVID-19 deaths in France and Ireland were from nursing homes, with even higher proportions reported in the US and Canada [5, 6], and high numbers of excess deaths in nursing homes reported in England and Wales [7].

Nursing homes provide care to the oldest-old in societies. Soon into the pandemic, advancing age was identified as a strong risk factor for COVID-19 mortality. In the US, it was reported that about 80% of deaths from COVID-19 were in individuals over the age of 65 [8]. In Spain, a study noted that while the overall infection fatality ratio for the general population was about 0.8%, it remained close to zero for individuals aged below 50 [9]. In addition, male sex was soon identified as a risk factor for COVID-19 mortality [9].

Beyond non-modifiable risk factors, such as residents’ age and sex, public and scientific discussions, soon followed considering if the high rate of dissemination and mortality in nursing homes could have also been related to potentially modifiable factors. Indeed, learnings on the latter could improve future pandemic preparedness. In the aftermath of the devastating impact of the COVID-19 pandemic on nursing homes, we undertook a scoping review of the literature to describe factors that contributed to the spread and mortality of COVID-19 in nursing homes and provided an overview of responses that were implemented to try to overcome such challenges.

## Methodology

### Step 1: literature search

We searched the MEDLINE Ovid database by combining the exploded MeSH subject headings (including all subheadings) and keywords “nursing homes” and “COVID-19” to generate an initial list of articles (all types). The time frame for the search was from 1st March 2020 to 31st January 2021. We limited the articles to English language. The MEDLINE Ovid search strategy was as follows:

**Table:** 

1	Nursing home.mp. or exp Home Nursing/
2	Covid-19.mp. or exp COVID-19/
3	1 and 2

### Step 2: study selection

Two reviewers (SG, LMC) independently screened article titles and abstracts, and then full articles that quantitatively or qualitatively identified factors that were associated with increased dissemination and mortality from COVID-19 in nursing homes, and strategies implemented to reduce them. Disagreements were resolved by involvement of a third reviewer (RRO) as necessary. Endnote^®^ software was used to prevent duplication of research articles.

### Step 3: reporting of the results

Data extracted from the included studies were categorised into the following themes: COVID-19 disease characteristics, resident-related factors, facility characteristics, staffing, and external factors. A Venn diagram of the number of articles in each theme was created with Lucidchart^®^ software. We also noted themes on responses for the mitigation of spread and mortality, including testing, isolation and cohorting, staff protection and support, promotion of residents’ physical and mental well-being, and technology in care.

## Results

**Fig. 1:**
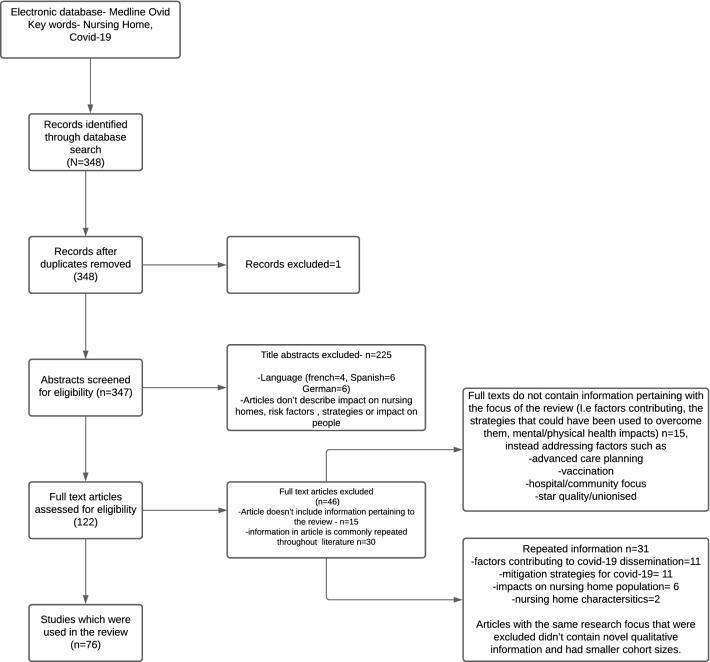
Flowchart of included studies

The flow chart of included studies is shown in Fig. 1.

**Fig. 2:**
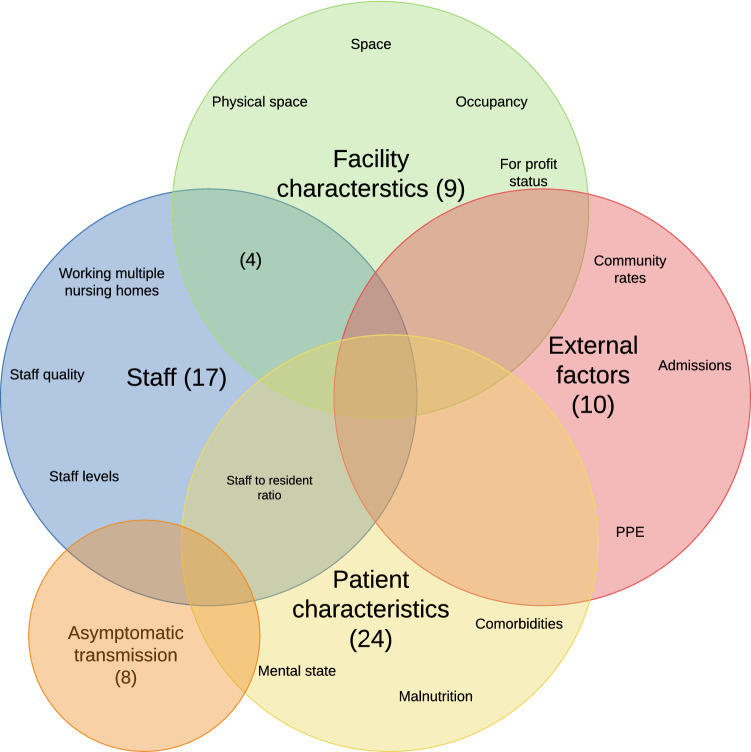
Venn diagram summarising factors associated with excess COVID-19 transmission and mortality in nursing homes. The number of included studies for each theme is in brackets. *PPE* personal protective equipment

The initial search retrieved 348 articles, of which 1 was a duplicate. Following the application of the exclusion criteria detailed in Fig. 1, 76 were included in the thematic review. 8 articles related to COVID-19 disease characteristics (asymptomatic transmission), 24 to resident-related factors (comorbidities, nutrition, cognition), 13 to facility characteristics (physical space, occupancy, for-profit status), 21 to staffing (staffing levels, staff-to-resident ratio, staff multi-employment), and 10 to external factors (community rates, availability of personal protective equipment, prevailing health and social care policies). Figure 2 shows the Venn diagram of those themes. In terms of responses, identified themes included widespread testing, isolation and cohorting of residents, staff protection, promotion of residents’ physical and mental well-being, and technological innovations. As reflected in Fig. 2, many included studies overlapped across different themes. When a study contained information on more than one theme, in the sections below we assigned the study to the main theme highlighted by the article.

### Residents’ characteristics

Nursing homes provide care and support to the most vulnerable members of society. Indeed, nursing home residents tend to be older, more disabled, and more cognitively impaired than people of comparable ages living in the community, and may have many other comorbidities contributing to their reliance on others for their care.

Specific comorbidities, such as cardiovascular diseases, diabetes, chronic respiratory disorders, hypertension, and cancer, have been linked to increased risk of COVID-19 mortality [10]. In a retrospective study of deceased COVID-19 patients in Germany, it was found that most patients had multiple comorbidities, with cardiovascular and respiratory diseases being the most common [11]. Aggravation of pre-existing cardiovascular conditions may explain why COVID-19 has been associated with high-risk acute cardiovascular events including myocardial infarction, myocarditis, cardiac arrhythmias, cardiogenic shock, thromboembolic disease, and cardiac arrest [12]. Pre-existing chronic respiratory conditions may increase the risk of a COVID-19 pneumonia becoming severe and life-threatening [13]. In addition, malnutrition is more prevalent in adults with comorbidities, being present in 24.6%, 23.3% and 12.9% of cognitively impaired, hypertensive, and diabetic older adults, respectively [14].

In nursing home residents who are living with dementia, their understanding of the necessary physical distancing measures and correct utilisation of personal protective equipment (PPE) may be limited, and this was also cited as a potential comorbidity-related mechanism contributing to COVID-19 spread in some facilities [15].

As well as comorbidity factors, other residents’ characteristics such as race/ethnicity may have also been connected to COVID-19 outcomes in nursing homes. In the US, higher dissemination rates were reported in nursing homes housing minority ethnic populations, with no clarity as to whether this could be driven by biological (e.g. racial susceptibility) or non-biological (e.g. socioeconomic, quality of care) factors [16–18].

### Facility characteristics

Higher number of beds with high occupancy within nursing homes may have also contributed to the large COVID-19 spread and mortality. Data from the US showed that smaller nursing homes were less likely to have a COVID-19 outbreak; however, once introduced, the dissemination of COVID-19 was often rapid, and related to factors including facility location and size, as well as other drivers detailed in the sections below [19, 20]. The regular influx of staff and visitors, and the high communal traffic, which are characteristic of these settings, may have further aggravated disease spread [21]. The urban location of many facilities, where community transmission rates were high, were associated with increased occurrence of nursing home outbreaks [22]. For-profit status was significantly associated with the extent and mortality of outbreaks [22]. Furthermore, nursing homes with open-layout designs and large rooms divided into smaller individual rooms by use of wooden screens may have contributed to outbreaks in Hong Kong nursing homes due to inadequate ventilation. In these homes, four-foot high wooden screens were used to divide whole floors into numerous single-person bedspaces [23].

### Staffing-related factors

Many nursing homes reported difficulties coping with the pandemic due to reduced staffing levels [24]. In the US, the pandemic aggravated a baseline situation where there was high staff turnover in nursing homes, with greater than 50% of nurses leaving their job within a year [25]. The devalued working conditions of nursing home staff meant that many workers held second jobs and double- or triple-duty caregiving roles [26]. In a London-based study, there was a significantly higher COVID-19 positivity among staff who worked at multiple facilities compared to staff working in a single facility [27]. In turn, the need for positive staff to self-isolate contributed to a ‘perfect storm’ of understaffing at many facilities [28].

Reports suggested that there was a greater probability of having COVID-19 resident infections in nursing homes where registered nurse levels were under the recommended minimum [20, 29, 30]. To emphasise the significance of this problem, a study suggested that a 20-min increase in registered staffing was associated with a 22% reduction in COVID-19 cases and 26% fewer COVID-19 deaths [31].

### External factors

In the US, it was argued that a tradition of low policy priority for the long-term care sector may have contributed to the relative unpreparedness and difficulties experienced by nursing homes in attempting to reduce COVID-19 transmission and mortality [32]. Furthermore, it was claimed that the declining financial support and supply to nursing homes may have led to reduced availability of PPE [32, 33]. Up to one in five nursing homes reported deficiencies in essential PPE supply [33]. In a study conducted in the US, 46% of facilities had a PPE shortage and/or a staffing shortage in at least 1 week over a 5-week study period, despite receiving state support for the procurement of PPE [34]. A study suggested that PPE shortages were more evident in private nursing homes compared to state-owned facilities [33]. This could be in relation to the different financial challenges experienced by these two different types of nursing homes [33], as well as external issues that generally affected the procurement and supply of PPE [35]. In the UK, it was argued that the primary PPE purchaser role of the NHS through the health care budget may have inadvertently undermined the procurement of PPE by nursing homes, which mostly depend on the social care budget and private income [36].

### COVID-19 characteristics: asymptomatic transmission

Asymptomatic transmission was paramount to the dissemination and mortality of COVID-19. Unsuspectedly, many nursing homes were found to have widespread dissemination levels due to a combination of atypical symptoms shown by residents and no symptoms shown by staff [37]. Proactive testing for COVID-19 among staff and residents showed high positivity proportions, making it obvious in retrospect that there had been wide unnoticed transmission of the virus [38]. A study in Spain showed that 69.7% of residents and 55.8% of staff had asymptomatic COVID-19 infection [39].

### Strategies to mitigate the impact of COVID-19 in nursing homes

To cope with COVID-19, a plethora of strategies were implemented in nursing homes. Due to the complexity of the pandemic, some of these strategies were borne of evidence while others were based on expert opinion or local initiatives. In some countries, the previous occurrence of other viral epidemics (e.g. SARS-CoV, MERS-CoV, H1N1) meant that there was a degree of understanding and preparedness surrounding mitigation of transmission in relation to PPE and sanitation [23]. However, for nursing homes in many other countries, COVID-19 represented their first learning curve.

### Widespread testing

The practice of testing for COVID-19 evolved throughout the pandemic. Initially, testing was only indicated in the presence of typical COVID-19 symptoms. However, it soon transpired that the prevalence of typical symptoms in nursing homes was much lower than expected, with cough and dyspnoea being present in only 30–60% of cases [40, 41], and a fever of over 38 degrees Celsius not being present in 70–77% of cases [42, 43]. To account for this, a cross-sectional study advocated for the lowering of the temperature threshold for the clinical suspicion of COVID-19 in nursing home residents [42], and another study suggested the measurement of basal temperature changes from the average as a better indicator of infection [43]. However, it soon became apparent that there was a significant failure to identify infection in residents when applying typical or modified symptoms criteria [39].

The subsequent rollout of widespread testing aided the identification of asymptomatic cases, which facilitated the rapid implementation of infection control procedures upon identification of infection. It was estimated that up to 50% of COVID-19 cases in nursing homes would have been missed without widespread testing [44]. Early studies reported decreases in COVID-19 transmission after the implementation of point prevalence surveys from 35 to 18% [45]. It was recommended that widespread testing should be conducted immediately after detection of a case, with delays in testing being associated with the risk of larger outbreaks [46].

### Isolation and cohorting

Given the variable time latencies between testing and results [24], and the need to repeat testing in many instances, isolation and cohorting strategies were implemented in nursing homes to divide the residents into defined groups based on COVID-19 status. Many studies reported the common practice of cohorting positive cases within a separate COVID-19 unit [47–49]. It was recommended that specific staff members be allocated to these units to reduce dissemination and allow for more focused care [50]. In addition, severe visiting restrictions were enforced to prevent external importation of the virus [51]. Many nursing homes implemented a 3-tier cohorting system (confirmed positive, suspected positive awaiting further testing, confirmed negative), as opposed to the 2-tier system used in previous influenza outbreaks [47]. In one study, 28% of exposed individuals who initially tested negative became positive shortly after [47]. This does not necessarily reflect poor sensitivity of the test, but the fact that it takes time after exposure, for any test to become positive as the infection becomes established and the virus excreted in enough quantity to be detectable by any test. In some facilities, cohorting strategies were difficult to implement due to lack of space and staff [50].

Later recommendations stated that residents should remain isolated for a full 14-day period after COVID-19 diagnosis unless having a required number of negative PCR tests [50]. However, the sensitivity of PCR tests was reported to drop after 10 days to 67% for nasal, and 47% for throat specimens [52].

Prolonged isolation predisposed residents to further physical and mental deconditioning [53]. The comorbidities associated with frail older residents, and especially dementia, often made isolation and cohorting very difficult. In many cases, the regular provision of information, reorientation, reassurance, and cognitive stimulation would have been effective [54], especially when trained staff were available [15]. However, more challenging scenarios arose in residents who developed superimposed hyperactive delirium and could not adhere to restrictions; in such cases, robust safeguarding policies had to be implemented centered on best interest and the use of the least restrictive options, to minimise instances of inappropriate physical and/or chemical restraints [54].

### Staff protection

Several studies highlighted high levels of dissatisfaction and anxiety among nursing home personnel, including financial, psychological, and work-related stressors [6, 55]. Many nursing home employees worked multiple jobs and, in some reports, up to 20% were worrying about their own food supplies [27, 56]. Nearly half of US states implemented policies to support staffing capacity and increase pay, to facilitate individuals to serve in their care roles [34]. Nursing homes that provided increased payments and incentives for staff demonstrated a 10% reduction in staff shortages [34]. It was noted that clear and coherent guidelines on isolation procedures could alleviate staff anxiety [6, 55], as well as holding regular multidisciplinary group discussions and staff debriefings [6, 50]. However, due to widespread staff shortages, many health care workers in nursing homes did not have the opportunity to avail themselves of their leave entitlements if they had them [27, 57]. One article advocated for additional social care workers who could support and maintain relationships with distressed family members, offsetting the burden of administrators and nurses [58].

One of the possible reasons for staff absenteeism was the fear of contracting the virus and/or transmitting it to a loved one, and adequate supplies of PPE helped address many of these fears [6]. Similarly, the introduction of national mask mandates led to a reduction in anxiety among health care workers [34]. Voluntary staff confinement in France showed that it protected residents from COVID-19 mortality and residents and staff from COVID-19 infection [59]. Daily testing of staff reduced both transmission among health care workers, and worker to patient transmission [52].

In the US, another type of staffing-related response was to try to increase the pool of available nursing home staff by allowing members of the public (e.g. the unemployed) to become temporary aides, and/or call medical reserve corps or public health service workers to volunteer [24]. Adjusted licensing/training requirements resulted in a 10% decrease in staff shortages, while long-term care job match assistance showed a 30% decrease in staff shortages [34]. Many of this newly sourced staff would have been trained to provide remote information and support to relatives and previous caregivers [60] and assist liaison with hospitals to ensure that the required transfer of care protocols were met [61].

### Technology in care

According to one study, the monitoring of residents’ vital signs via telehealth was feasible [62], but this is not available in most nursing homes. Technology also attempted to provide an alternative means of tracking dissemination [3]. Such a system could reduce the burden on manual contact tracers and facilitate a faster means of identifying infections [63]. In a nursing home setting, where only 17% of adults over the age of 80 use a phone, wearable devices would perhaps be a more suitable option [3]. Robotic support for the care of nursing home residents is also possible [64], but sporadically available.

### Physical and mental health support for residents

During the pandemic, reduced staff-resident contact time, lower levels of physical activity, decreased mealtime conversation and reduced social interactions may all have contributed to physical decline and/or weight loss in some residents [65], new instances of pressure ulcers and/or falls [20], and a general decline in psychological well-being and cognition, necessitating new supports for residents [66–69] that were not previously mandated by quality standards [70]. Many improvement initiatives focused on non-pharmacological measures including cognitive stimulation, nutritional optimisations, increased walking, and trying to maintain social interaction [67]. Studies suggested that poor mental health outcomes associated with lockdown can be mitigated with thoughtful intervention and ongoing evaluation with clinical information systems [71–73]. The European Geriatric Medicine Society advocated for the use of a programme known as MATCH (maintenance of autonomy through exercise care during hospitalisation) involving the use of a decision tree to cater to the various fitness levels of residents [50].

To maintain social connection, numerous methods were observed, for example via window or behind-glass visiting [23]. Video calls alongside letters and telephone calls were reported to aid in reducing depression among nursing home residents, and were also associated with positive social interaction, decreased loneliness, and better quality of life [74, 75]. Some video-calling modalities posed problems for older users, including auditory interference and a lack of familiarity with the technology [76], highlighting the need for the development of technology that is specifically tailored to older people. Another technology known as simulated presence therapy, whereby video messages are frequently exchanged between family and the older person, led to the enhanced well-being of residents living with dementia and decreased behavioural and psychological symptoms [77]. Qualitative positive feedback was observed compared with traditional telephone communication [78]. Cognitive behavioural therapy and mindfulness could be implemented in some facilities with positive results [74].

## Discussion

The COVID-19 pandemic had multiple negative impacts in nursing homes. Our literature review highlighted numerous ‘frailties’ in this sector that contributed to poor outcomes, but we also identified multiple responses that successfully mitigated some of the adverse impacts. Multiple factors simultaneously contributed to the challenges including disease characteristics (asymptomatic transmission), resident-related factors (comorbidities, nutrition, cognition), facility characteristics (physical space, occupancy, for-profit status), staffing (staffing levels, staff-to-resident ratio, staff multi-employment), and external factors (availability of personal protective equipment, prevailing health and social care policies). In terms of responses, identified themes included widespread testing, isolation and cohorting of residents, staff protection and support, promotion of residents’ well-being, and technological innovations.

Outbreaks did not affect all facilities equally. A variety of internal and external factors interacted in each case to produce outbreaks of varying magnitude and severity. Some of these factors may be unmodifiable in the short term, including the characteristics of the virus, and many facility and patient characteristics. On the other hand, many staff and external factors were rapidly actioned upon. Yet even upon recognition of these modifiable risks, difficulties in modifying these factors, in combination with a virus which refused to relent, meant that outbreaks continued to occur. Early in the pandemic, a degree of trial and error may have been required to determine the most suitable alternative, as was seen in the implementation of testing and isolation procedures.

This review used a well-defined search strategy to select qualitative and quantitative aspects of the effects and management of COVID-19 in nursing homes. However, we acknowledge certain methodological limitations. First, published literature was only limited to one bibliographical database (PubMed); thus, some relevant articles not indexed in PubMed, or existing in the grey literature, may have been excluded. Second, most included studies were observational and often describing single-centre experiences. Hence, further research is required to elucidate the effectiveness and feasibility of many of the interventions identified in this review. Furthermore, we did not consider articles in languages other than English. The scoping nature of our review and a limit on the maximum number of references that we could include meant that only 76 articles were selected from 347 identified by the literature search. Our limited search strategy may have excluded studies specifically related to ventilation or airborne transmission routes with nursing homes, including entry/exit or circulation, single versus shared rooms, or other built environment related issues. In addition, we may have not captured studies looking at staff movement between units or floors within a facility. Another limitation is that using the MeSH term ‘nursing homes’ and exploding it, we included related descriptors, such as skilled nursing facilities and intermediate care facilities; however, some relevant descriptors for these facilities may have been excluded.

Nursing homes underwent a steep learning curve during the COVID-19 pandemic. With the lessons learnt, new pandemic preparedness plans can be created for every nursing home, incorporating staff reorganisation, PPE procurement strategies, and effective space allocation plans. Being prepared for the future is more than just about having a plan: it is about fostering the infrastructure and culture that can help respond to events of such a scale. Staff protection, support and education are paramount to this. A plan for delegation of tasks in such a scenario, with some individuals responsible for communication with family members and others for ward-specific tasks, might be helpful. Many nursing homes will invest in new technologies that will help preparedness. Evidence-based guidelines surrounding implementation of these technologies in nursing homes should be created, ensuring that resources have a positive effect on user quality of life and that staff are trained appropriately in their use.

## Conclusion

COVID-19 has exerted a devastating effect on the nursing home population and its staff, significantly affecting their mental and physical health. A myriad of interacting factors in nursing homes predisposed them to an increased propensity of spread, some non-modifiable and some potentially modifiable. Given that facility characteristics emerged as one of the key themes in this review, considerations should be given to the future design or adaptation of nursing home facilities. In the aftermath of the pandemic, we can change built environments, conventions, cultures, and policies in the nursing home sector, not just to prepare for future pandemics, but to improve the overall quality of care offered at all times. It is essential that these lessons are learnt so that nursing homes and their residents may have a better future.

## Data Availability

N/A.

## Code availability

N/A.

## References

[1] Grabowski DC, Mor V (2020). Nursing home care in crisis in the wake of COVID-19. JAMA.

[2] Beland D, Marier P (2020). COVID-19 and long-term care policy for older people in Canada. J Aging Soc Policy.

[3] Wilmink G, Summer I, Marsyla D, Sukhu S, Grote J, Zobel G (2020). Real-time digital contact tracing: development of a system to control COVID-19 outbreaks in nursing homes and long-term care facilities. JMIR Public Health Surveill.

[4] O'Neill D, Briggs R, Holmerova I, Samuelsson O, Gordon AL, Martin FC (2020). COVID-19 highlights the need for universal adoption of standards of medical care for physicians in nursing homes in Europe. Eur Geriatr Med.

[5] Iritani O, Okuno T, Hama D, Kane A, Kodera K, Morigaki K (2020). Clusters of COVID-19 in long-term care hospitals and facilities in Japan from 16 January to 9 May 2020. Geriatr Gerontol Int.

[6] McGilton KS, Escrig-Pinol A, Gordon A, Chu CH, Zuniga F, Sanchez MG (2020). Uncovering the devaluation of nursing home staff during COVID-19: are we fuelling the next health care crisis?. J Am Med Dir Assoc.

[7] Burki T (2020). England and Wales see 20 000 excess deaths in care homes. Lancet.

[8] Powell T, Bellin E, Ehrlich AR (2020). Older adults and COVID-19: the most vulnerable, the hardest hit. Hastings Cent Rep.

[9] Mallapaty S (2020). The coronavirus is most deadly if you are older and male—new data reveal the risks. Nature.

[10] Dosa D, Jump RLP, LaPlante K, Gravenstein S (2020). Long-term care facilities and the coronavirus epidemic: practical guidelines for a population at highest risk. J Am Med Dir Assoc.

[11] Edler C, Schroder AS, Aepfelbacher M, Fitzek A, Heinemann A, Heinrich F (2020). Dying with SARS-CoV-2 infection-an autopsy study of the first consecutive 80 cases in Hamburg, Germany. Int J Legal Med.

[12] Nabors C, Sridhar A, Hooda U, Lobo SA, Levine A, Frishman WH (2021). Characteristics and outcomes of patients 80 years and older hospitalized with coronavirus disease 2019 (COVID-19). Cardiol Rev.

[13] Sacco G, Foucault G, Briere O, Annweiler C (2020). COVID-19 in seniors: findings and lessons from mass screening in a nursing home. Maturitas.

[14] Araujo MPD, Nunes VMA, Costa LA, Souza TA, Torres GV, Nobre TTX (2021). Health conditions of potential risk for severe COVID-19 in institutionalized elderly people. PLoS ONE.

[15] Ryoo N, Pyun JM, Baek MJ, Suh J, Kang MJ, Wang MJ (2020). Coping with dementia in the middle of the COVID-19 pandemic. J Korean Med Sci.

[16] Sugg MM, Spaulding TJ, Lane SJ, Runkle JD, Harden SR, Hege A (2021). Mapping community-level determinants of COVID-19 transmission in nursing homes: a multi-scale approach. Sci Total Environ.

[17] Shippee TP, Akosionu O, Ng W, Woodhouse M, Duan Y, Thao MS (2020). COVID-19 pandemic: exacerbating racial/ethnic disparities in long-term services and supports. J Aging Soc Policy.

[18] Lipsitz LA, Lujan AM, Dufour A, Abrahams G, Magliozzi H, Herndon L (2020). Stemming the tide of COVID-19 infections in Massachusetts nursing homes. J Am Geriatr Soc.

[19] Abrams HR, Loomer L, Gandhi A, Grabowski DC (2020). Characteristics of U.S. nursing homes with COVID-19 cases. J Am Geriatr Soc.

[20] Harrington C, Ross L, Chapman S, Halifax E, Spurlock B, Bakerjian D (2020). Nurse staffing and coronavirus infections in California nursing homes. Policy Polit Nurs Pract.

[21] Anderson DC, Grey T, Kennelly S, O'Neill D (2020). Nursing home design and COVID-19: balancing infection control, quality of life, and resilience. J Am Med Dir Assoc.

[22] Stall NM, Jones A, Brown KA, Rochon PA, Costa AP (2020). For-profit long-term care homes and the risk of COVID-19 outbreaks and resident deaths. CMAJ.

[23] Chow L (2021). Care homes and COVID-19 in Hong Kong: how the lessons from SARS were used to good effect. Age Ageing.

[24] Abbasi J (2020). "Abandoned" nursing homes continue to face critical supply and staff shortages as COVID-19 toll has mounted. JAMA.

[25] Chen AT, Ryskina KL, Jung HY (2020). Long-term care, residential facilities, and COVID-19: an overview of federal and state policy responses. J Am Med Dir Assoc.

[26] Van Houtven CH, DePasquale N, Coe NB (2020). Essential long-term care workers Commonly hold second jobs and double- or triple-duty caregiving roles. J Am Geriatr Soc.

[27] Ladhani SN, Chow JY, Janarthanan R, Fok J, Crawley-Boevey E, Vusirikala A (2020). Increased risk of SARS-CoV-2 infection in staff working across different care homes: enhanced COVID-19 outbreak investigations in London care Homes. J Infect.

[28] Ouslander JG, Grabowski DC (2020). COVID-19 in nursing homes: calming the perfect storm. J Am Geriatr Soc.

[29] Fallon A, Dukelow T, Kennelly SP, O'Neill D (2020). COVID-19 in nursing homes. QJM.

[30] Leskovic L, Erjavec K, Leskovar R, Vukovic G (2020). Burnout and job satisfaction of healthcare workers in Slovenian nursing homes in rural areas during the COVID-19 pandemic. Ann Agric Environ Med.

[31] Li Y, Temkin-Greener H, Shan G, Cai X (2020). COVID-19 infections and deaths among Connecticut nursing home residents: facility correlates. J Am Geriatr Soc.

[32] Werner RM, Hoffman AK, Coe NB (2020). Long-term care policy after COVID-19—solving the nursing home crisis. N Engl J Med.

[33] McGarry BE, Grabowski DC, Barnett ML (2020). Severe staffing and personal protective equipment shortages faced by nursing homes during the COVID-19 pandemic. Health Aff (Millwood).

[34] Gibson DM, Greene J (2020). State actions and shortages of personal protective equipment and staff in U.S. nursing homes. J Am Geriatr Soc.

[35] Dini FL, Bergamini C, Allegrini A, Scopelliti M, Secco G, Miccoli M (2020). Bedside wireless lung ultrasound for the evaluation of COVID-19 lung injury in senior nursing home residents. Monaldi Arch Chest Dis.

[36] Gordon AL, Goodman C, Achterberg W, Barker RO, Burns E, Hanratty B (2020). Commentary: COVID in care homes-challenges and dilemmas in healthcare delivery. Age Ageing.

[37] Kittang BR, Hofacker SV, Solheim SP, Kruger K, Loland KK, Jansen K (2020). Outbreak of COVID-19 at three nursing homes in Bergen. Tidsskr Nor Laegeforen.

[38] Graham NSN, Junghans C, Downes R, Sendall C, Lai H, McKirdy A (2020). SARS-CoV-2 infection, clinical features and outcome of COVID-19 in United Kingdom nursing homes. J Infect.

[39] Borras-Bermejo B, Martinez-Gomez X, San Miguel MG, Esperalba J, Anton A, Martin E (2020). Asymptomatic SARS-CoV-2 infection in nursing homes, Barcelona, Spain, April 2020. Emerg Infect Dis.

[40] Blain H, Rolland Y, Benetos A, Giacosa N, Albrand M, Miot S (2020). Atypical clinical presentation of COVID-19 infection in residents of a long-term care facility. Eur Geriatr Med.

[41] Rutten JJS, van Loon AM, van Kooten J, van Buul LW, Joling KJ, Smalbrugge M (2020). Clinical suspicion of COVID-19 in nursing home residents: symptoms and mortality risk factors. J Am Med Dir Assoc.

[42] McConeghy KW, White E, Panagiotou OA, Santostefano C, Halladay C, Feifer RA (2020). Temperature screening for SARS-CoV-2 in nursing homes: evidence from two national cohorts. J Am Geriatr Soc.

[43] Rudolph JL, Halladay CW, Barber M, McConeghy KW, Mor V, Nanda A (2020). Temperature in nursing home residents systematically tested for SARS-CoV-2. J Am Med Dir Assoc.

[44] Birgand G, Blanckaert K, Deschanvres C, Vaudron A, Loury P, King L (2021). Testing strategies for the control of COVID-19 in nursing homes: Universal or targeted screening?. J Infect.

[45] Sanchez GV, Biedron C, Fink LR, Hatfield KM, Polistico JMF, Meyer MP (2020). Initial and repeated point prevalence surveys to inform SARS-CoV-2 infection prevention in 26 skilled nursing facilities—Detroit, Michigan, March-May 2020. MMWR Morb Mortal Wkly Rep.

[46] Blackman C, Farber S, Feifer RA, Mor V, White EM (2020). An illustration of SARS-CoV-2 dissemination within a skilled nursing facility using heat maps. J Am Geriatr Soc.

[47] Collison M, Beiting KJ, Walker J, Huisingh-Scheetz M, Pisano J, Chia S (2020). Three-tiered COVID-19 cohorting strategy and implications for memory-care. J Am Med Dir Assoc.

[48] Gonzalez de Villaumbrosia C, Martinez Peromingo J, Ortiz Imedio J, Alvarez Espejo de Montiel T, Garcia-Puente Suarez L, Navas Clemente I (2020). Implementation of an algorithm of cohort classification to prevent the spread of COVID-19 in nursing homes. J Am Med Dir Assoc.

[49] Krone M, Noffz A, Richter E, Vogel U, Schwab M (2021). Control of a COVID-19 outbreak in a nursing home by general screening and cohort isolation in Germany, March to May 2020. Euro Surveill.

[50] Blain H, Rolland Y, Schols J, Cherubini A, Miot S, O'Neill D (2020). Interim EuGMS guidance to prepare European long-term care facilities for COVID-19. Eur Geriatr Med.

[51] Khan AA, Singh VP, Khan D (2020). The care home pandemic—what lessons can we learn for the future?. J Gerontol Soc Work.

[52] Yates TA, Cooke GS, MacPherson P (2020). Rational use of SARS-CoV-2 polymerase chain reaction tests within institutions caring for the vulnerable. F1000Res.

[53] Iaboni A, Cockburn A, Marcil M, Rodrigues K, Marshall C, Garcia MA (2020). Achieving safe, effective, and compassionate quarantine or isolation of older adults with Dementia in nursing homes. Am J Geriatr Psychiatry.

[54] Liddell K, Ruck Keene A, Holland A, Huppert J, Underwood BR, Clark O (2021). Isolating residents including wandering residents in care and group homes: medical ethics and English law in the context of COVID-19. Int J Law Psychiatry.

[55] Senczyszyn A, Lion KM, Szczesniak D, Trypka E, Mazurek J, Ciulkowicz M (2020). Mental health impact of SARS-COV-2 pandemic on long-term care facility personnel in Poland. J Am Med Dir Assoc.

[56] Buccafusca M, Micali C, Autunno M, Versace AG, Nunnari G, Musumeci O (2021). Favourable course in a cohort of Parkinson's disease patients infected by SARS-CoV-2: a single-centre experience. Neurol Sci.

[57] Greene J, Gibson DM (2021). Workers at long-term care facilities and their risk for severe COVID-19 illness. Prev Med.

[58] Bern-Klug M, Beaulieu E (2020). COVID-19 highlights the need for trained social workers in nursing homes. J Am Med Dir Assoc.

[59] Belmin J, Um-Din N, Donadio C, Magri M, Nghiem QD, Oquendo B, Disease C (2019). Outcomes in French nursing homes that implemented staff confinement with residents. JAMA Netw Open.

[60] Gonzalez-Fraile E, Ballesteros J, Rueda JR, Santos-Zorrozua B, Sola I, McCleery J (2021). Remotely delivered information, training and support for informal caregivers of people with dementia. Cochrane Database Syst Rev.

[61] Sze S, Pan D, Williams CML, Barker J, Minhas JS, Miller CJ (2021). The need for improved discharge criteria for hospitalised patients with COVID-19-implications for patients in long-term care facilities. Age Ageing.

[62] Ohligs M, Stocklassa S, Rossaint R, Czaplik M, Follmann A (2020). Employment of telemedicine in nursing homes: clinical requirement analysis, system development and first test results. Clin Interv Aging.

[63] Echeverria P, Mas Bergas MA, Puig J, Isnard M, Massot M, Vedia C (2020). COVIDApp as an innovative strategy for the management and follow-up of COVID-19 cases in long-term care facilities in Catalonia: implementation study. JMIR Public Health Surveill.

[64] Lanza F, Seidita V, Chella A (2020). Agents and robots for collaborating and supporting physicians in healthcare scenarios. J Biomed Inform.

[65] Danilovich MK, Norrick CR, Hill KC, Conroy DE (2020). Nursing home resident weight loss during coronavirus disease 2019 restrictions. J Am Med Dir Assoc.

[66] Havaei F, Macphee M, Keselman D, Staempfli S (2021). Leading a long-term care facility through the COVID-19 crisis: successes, barriers and lessons learned. Healthc Q.

[67] Canevelli M, Bruno G, Cesari M (2020). Providing simultaneous COVID-19-sensitive and dementia-sensitive care as we transition from crisis care to ongoing care. J Am Med Dir Assoc.

[68] Flatharta TO, Mulkerrin EC (2020). Back to basics: Giant Challenges to addressing Isaac's "Geriatric Giants" post COVID-19 crisis. J Nutr Health Aging.

[69] Aubertin-Leheudre M, Rolland Y (2020). The importance of physical activity to care for frail older adults during the COVID-19 pandemic. J Am Med Dir Assoc.

[70] Bui DP, See I, Hesse EM, Varela K, Harvey RR, August EM (2020). Association between CMS quality ratings and COVID-19 outbreaks in nursing homes—West Virginia, March 17-June 11, 2020. MMWR Morb Mortal Wkly Rep.

[71] Bacsu JD, O'Connell ME, Cammer A, Azizi M, Grewal K, Poole L (2021). Using twitter to understand the COVID-19 experiences of people with Dementia: infodemiology study. J Med Internet Res.

[72] Robison J, Shugrue N, Migneault D, Charles D, Baker K, Fortinsky R (2021). Community-based long-term care has lower COVID-19 rates and improved outcomes compared to residential settings. J Am Med Dir Assoc.

[73] McArthur C, Saari M, Heckman GA, Wellens N, Weir J, Hebert P (2021). Evaluating the effect of covid-19 pandemic lockdown on long-term care residents' mental health: a data-driven approach in New Brunswick. J Am Med Dir Assoc.

[74] Gorenko JA, Moran C, Flynn M, Dobson K, Konnert C (2021). Social isolation and psychological distress among older adults related to COVID-19: a narrative review of remotely-delivered interventions and recommendations. J Appl Gerontol.

[75] Noone C, McSharry J, Smalle M, Burns A, Dwan K, Devane D (2020). Video calls for reducing social isolation and loneliness in older people: a rapid review. Cochrane Database Syst Rev.

[76] Chu CH, Donato-Woodger S, Dainton CJ (2020). Competing crises: COVID-19 countermeasures and social isolation among older adults in long-term care. J Adv Nurs.

[77] Simard J, Volicer L (2020). Loneliness and isolation in long-term care and the COVID-19 pandemic. J Am Med Dir Assoc.

[78] van Dyck LI, Wilkins KM, Ouellet J, Ouellet GM, Conroy ML (2020). Combating heightened social isolation of nursing home elders: the telephone outreach in the COVID-19 outbreak program. Am J Geriatr Psychiatry.

